# Comparative analysis and optimization of tumor regression grade assessment systems in neoadjuvant therapy for esophageal squamous cell carcinoma

**DOI:** 10.1186/s12885-025-14726-4

**Published:** 2025-08-20

**Authors:** Qiao Yang, Xiaoqing Zhou, Jiayu Li, Jieyu Wang, Jingcheng Luo, Dan Luo, Lin Shi, Chengmin Zhou, Yi Wang, Xuefeng Leng, Qifeng Wang, Yang Liu, Yehan Zhou

**Affiliations:** Department of Pathology, Sichuan Cancer Center, School of Medicine, Sichuan Cancer Hospital & Institute, University of Electronic Science and Technology of China, Chengdu, Sichuan China

**Keywords:** Tumor regression grade, Esophageal cancer, Prognosis, Lymph nodes

## Abstract

**Objective:**

This study aims to compare and analyze the effectiveness of different tumor regression grade (TRG) assessment systems in evaluating neoadjuvant therapy for esophageal cancer, with the goal of identifying an optimal assessment method to guide clinical practice.

**Methods:**

A total of 467 patients with esophageal squamous cell carcinoma who underwent neoadjuvant therapy followed by surgical resection were included in this study. We comprehensively evaluated the effectiveness of five TRG assessment systems—Mandard, CAP, Becker, JSED, and Ryan—in predicting the prognosis of the primary tumor (PT) and lymph nodes (LN). The inter-observer consistency among these systems was also assessed to identify the most effective TRG evaluation method.

**Results:**

The performance of the TRG assessment systems in predicting LN prognosis was generally superior to that for PT. Specifically, while the Ryan criteria demonstrated the highest inter-observer consistency coefficient (Mean Kappa = 0.848), its predictive efficacy was the lowest (Mean AUC = 0.502). In contrast, the Becker criteria exhibited the highest predictive efficacy (Mean AUC = 0.609) alongside a good consistency coefficient (Mean Kappa = 0.788). Notably, the modified Modified TRG system not only achieved a higher AUC value (0.624) but also showed excellent inter-observer consistency (Kappa = 0.904).

**Conclusion:**

The modified Modified TRG system, with a focus on LN evaluation, demonstrates superior prognostic predictive ability and risk stratification effectiveness. These findings may assist clinicians in more accurately assessing patient prognosis and adjusting treatment strategies accordingly, ultimately optimizing patient treatment pathways.

**Supplementary Information:**

The online version contains supplementary material available at 10.1186/s12885-025-14726-4.

## Introduction

Esophageal cancer has a high incidence and mortality rate globally, with China accounting for nearly half of the cases. As a highly lethal malignancy, it ranks fourth among cancer-related deaths, posing a serious threat to public health. Many patients in China are diagnosed at advanced stages, resulting in poor prognoses. The emergence of neoadjuvant treatment strategies offers new hope for improving survival and outcomes in these patients. However, the traditional TNM staging system has limitations in predicting patient prognosis, highlighting the need for more effective assessment tools.

In this context, the Tumor Regression Grade (TRG) evaluation system has emerged, proving to be a valuable prognostic indicator for esophageal cancer in multiple high-quality studies. This system allows for precise risk stratification based on the tumor’s response to treatment, providing a scientific basis for doctors to formulate more personalized treatment strategies. Thus, it promotes the precision and individualization of esophageal cancer treatment decisions.

Nevertheless, the evaluation of TRG faces numerous complex and severe challenges. Firstly, there is a wide variety of assessment systems, and significant discrepancies exist among different standards, along with a lack of unified consensus [1, 2]. Researchers such as Mandard and Backer have delved into the application value of their respective standards in prognostic assessments of gastrointestinal tumors, confirming the stratification ability of each [3–7]. However, which standard is most suitable for esophageal cancer remains undetermined. Additionally, it is noteworthy that the predominant histological type of esophageal cancer in Western populations is adenocarcinoma, whereas squamous cell carcinoma predominates in China. Consequently, findings based on Western population data may face limitations in applicability and accuracy when applied to Chinese patients. This not only complicates the evaluation systems but also emphasizes the urgency of finding a TRG assessment system suited for the Chinese population.

Moreover, even under the same assessment standard, subjective differences in observer judgments often lead to inconsistencies in evaluation results, undoubtedly increasing the difficulty and risk of clinical decision-making [8]. More challenging is the fact that current TRG evaluations primarily focus on the primary lesion, while lacking clear and unified assessment standards for lymph node metastasis, a key prognostic factor [9]. This situation not only limits the widespread application of the system in clinical practice but also hinders further optimization of precision treatment strategies for esophageal cancer.

In light of these challenges, this study aims to explore and compare the assessment efficacy and inter-observer consistency of commonly used TRG methods among esophageal cancer patients at our center. By employing a combination of statistical analysis and clinical validation, we hope to provide a more precise and reliable basis for prognostic assessment in esophageal cancer patients. Ultimately, this will guide clinical decision-making, optimize treatment plans, improve patients’ quality of life, and extend survival periods. This study not only holds significant clinical importance but also offers valuable insights for optimizing precision treatment strategies for esophageal cancer.

## Study subjects and methods

### Study subjects

This study included patients with esophageal cancer treated at Sichuan Cancer Hospital between January 2018 and January 2022. Inclusion criteria were strictly defined as follows: (1) diagnosis of primary locally advanced esophageal squamous cell carcinoma; (2) absence of concurrent malignant tumors; (3) receipt of preoperative neoadjuvant therapy, consisting of radiotherapy and chemotherapy or chemotherapy combined with immunotherapy; (4) subsequent radical esophagectomy following neoadjuvant therapy; (5) no clinical evidence of distant metastasis; (6) no history of autoimmune diseases; and (7) complete clinical data. Following a rigorous screening process, a total of 467 patients were enrolled(Supplementary Fig. 1).

In terms of preoperative neoadjuvant therapy, all patients received chemotherapy, with a mean of 1.99 ± 0.52 cycles. The chemotherapeutic agents included albumin-bound paclitaxel, carboplatin, cisplatin, docetaxel, oxaliplatin, and fluorouracil, administered either as monotherapy or in combination. Among these, 282 patients also received image-guided intensity-modulated radiotherapy (IMRT) during the neoadjuvant therapy period. The radiation doses were as follows: primary tumor gross target volume (GTV) at 2.0 Gy per fraction, left lymph node target volume (GTVInL) at 2.0 Gy per fraction, right lymph node target volume (GTVInR) at 2.0 Gy per fraction, and clinical target volume (CTV) at 1.8 Gy per fraction, with a total of 20.32 ± 1.14 sessions, accumulating to a total radiation dose of 40.49 ± 1.75 Gy. Additionally, 185 patients received immunotherapy during neoadjuvant treatment, with agents including nivolumab, pembrolizumab, and sintilimab.

## Methods

All patients underwent radical esophagectomy with two-field (thoraco-abdominal) or three-field (cervico-thoraco-abdominal) lymphadenectomy within 4–6 weeks after completing neoadjuvant therapy. Procedures were performed by experienced surgeons (> 50 esophagectomies/year) under standardized protocols: Surgical Approach Selection.Transthoracic Esophagectomy (Ivor Lewis): Abdominal phase: Upper midline incision; gastric mobilization preserving right gastroepiploic arcade.Thoracic phase: Right posterolateral thoracotomy (5th ICS) or VATS/RATS (4-port); *en bloc* resection of esophagus, periesophageal tissue, and mediastinal lymph nodes.McKeown Approach (3-field): Added cervical phase: Left cervical incision (along sternocleidomastoid); dissection of recurrent laryngeal nerve (RLN) basins.Lymphadenectomy Extent:2-field:*Abdomen*: Stations 1, 2, 3, 7, 8a, 9, 11p.*Thorax*: Stations 107, 108, 109, 110, 111, 112.3-field: Additional *cervical*: Stations 101, 102, 104.Critical Technical Elements: Recurrent Laryngeal Nerve Protection: Intraoperative neuromonitoring (IONM) during cervical/upper mediastinal dissection.Anastomotic Technique: Cervical: Hand-sewn end-to-end (2-layer interrupted 4 − 0 PDS).Intrathoracic: Circular stapler (25–28 mm) + reinforced suture.Conduit Reconstruction: Gastric tube (4–5 cm width) routed through posterior mediastinum.Pyloric drainage (pyloromyotomy/pyloroplasty). Quality Control Measures: Minimum lymph node harvest: ≥15 (2-field) or ≥ 25 (3-field). Intraoperative frozen section for proximal/distal margins.RLN nodal basins tagged separately for pathology.

For specimen processing, this study employed standard histological methods. Specifically, surgically resected tumor specimens were fixed in 10% neutral formalin, followed by paraffin embedding to create conventional sections. The sections were then stained with hematoxylin and eosin for subsequent evaluation of tumor regression grading.

The assessment of tumor regression grading (TRG) referenced five commonly used clinical grading systems: Mandard, CAP, Becker, JSED, and Ryan [3–7] (Table 1). These systems primarily grade based on the quantity of surviving tumor cells relative to the stroma. During the evaluation process, two experienced pathologists meticulously examined and assessed the slides. In cases of disagreement, P40 and PCK immunohistochemical staining were utilized, and a third senior pathologist reviewed the results to ensure the accuracy and reliability of the evaluations.

**Table 1:** Various types of TRG assessment criteria

Mandard	CAP	Becker	JSED	Ryan
TRG1 No residual cancer cells	TRG0 No residual cancer cells	TRG1a Complete regression	TRG1 0% residual tumor	TRG1 No or rare residual cancer cells
TRG2 Rare cancer cells	TRG1 Single cell or small group of cells	TRG1b <10% residual tumor	TRG2 1–33% residual tumor	TRG2 More residual cancer cells, but outgrowing by fibrosis
TRG3 Fibrosis outgrowing residual cancer	TRG2 Residual cancer with desmoplastic response	TRG2 10–50% residual tumor	TRG3 34–66% residual tumor	TRG3 Residual cancer cells outgrowing fibrosis or no regression
TRG4 Residual cancer outgrowing fibrosis	TRG3 Minimal evidence of tumor response	TRG3 >50% residual tumor	TRG4 67–100% residual tumor	
TRG5 Absence of regressive change				

*CAP*  College of American Pathologists, *JSED*   Japanese Society of Esophageal Diseases

### Study subjects

This study included patients with esophageal cancer treated at Sichuan Cancer Hospital between January 2018 and January 2022. Inclusion criteria were strictly defined as follows: (1) diagnosis of primary locally advanced esophageal squamous cell carcinoma; (2) absence of concurrent malignant tumors; (3) receipt of preoperative neoadjuvant therapy, consisting of radiotherapy and chemotherapy or chemotherapy combined with immunotherapy; (4) subsequent radical esophagectomy following neoadjuvant therapy; (5) no clinical evidence of distant metastasis; (6) no history of autoimmune diseases; and (7) complete clinical data. Following a rigorous screening process, a total of 467 patients were enrolled(Supplementary Fig. 1).

In terms of preoperative neoadjuvant therapy, all patients received chemotherapy, with a mean of 1.99 ± 0.52 cycles. The chemotherapeutic agents included albumin-bound paclitaxel, carboplatin, cisplatin, docetaxel, oxaliplatin, and fluorouracil, administered either as monotherapy or in combination. Among these, 282 patients also received image-guided intensity-modulated radiotherapy (IMRT) during the neoadjuvant therapy period. The radiation doses were as follows: primary tumor gross target volume (GTV) at 2.0 Gy per fraction, left lymph node target volume (GTVInL) at 2.0 Gy per fraction, right lymph node target volume (GTVInR) at 2.0 Gy per fraction, and clinical target volume (CTV) at 1.8 Gy per fraction, with a total of 20.32 ± 1.14 sessions, accumulating to a total radiation dose of 40.49 ± 1.75 Gy. Additionally, 185 patients received immunotherapy during neoadjuvant treatment, with agents including nivolumab, pembrolizumab, and sintilimab.

## Results

**Fig. 1:**
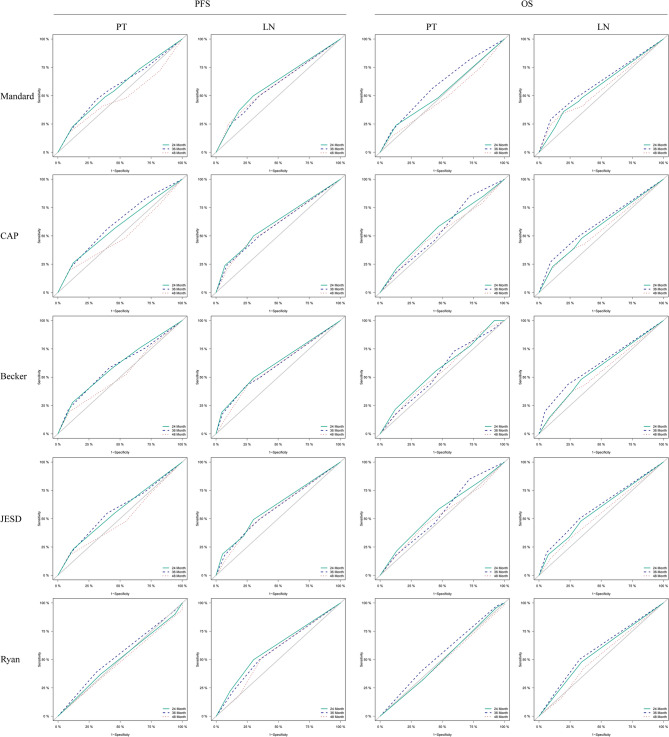
Assessment of the effectiveness of different tumor regression grade (TRG) standards applied to primary tumors (PT) and lymph nodes (LN) in evaluating patient prognosis, specifically regarding progression-free survival (PFS) and overall survival (OS), using receiver operating characteristic (ROC) curves

This study adopted five widely used tumor regression grading (TRG) evaluation criteria in clinical practice, conducting a systematic assessment of both primary tumors (PT) and lymph nodes (LN) to accurately predict patients’ overall survival (OS) and progression-free survival (PFS) risks. A detailed comparison of the area under the ROC curve (AUC) revealed that the overall prognostic predictive efficacy of LN-TRG (Mean AUC = 0.594) was superior to that of PT-TRG (Mean AUC = 0.545). Furthermore, both PT-TRG and LN-TRG demonstrated greater predictive efficacy for PFS compared to OS. Notably, the efficacy of PT-TRG in predicting 4-year PFS (AUC = 0.494) showed a significant decline compared to the predictions for 2-year and 3-year PFS (AUCs of 0.564 and 0.577, respectively). During the evaluation process of LN and PT using TRG criteria, we observed that the inter-observer consistency coefficient Kappa for LN assessment (0.804) was significantly higher than that for PT assessment (0.785). Further analysis of the five assessment criteria revealed that, although the Ryan criterion exhibited the highest consistency coefficient (0.848), its predictive efficacy was the lowest (AUC = 0.474), while the Becker criterion displayed a higher predictive efficacy (AUC = 0.609) along with a good consistency coefficient (0.816) (Fig. 1; Table 2). This analysis of 467 esophageal cancer patients receiving neoadjuvant therapy (NCRT = 282, NICT = 185) revealed comparable baseline demographics (sex, age, smoking, drinking; all *p* > 0.05). However, pretreatment clinical staging differed significantly, with the NICT group having higher cT3 rates (91.9% vs. 78.0%, *p* < 0.001) and the NCRT group containing cT4b cases (7.1% vs. 0%, *p* < 0.001). Pathological assessment post-treatment demonstrated superior tumor response in the NCRT group: higher rates of complete pathological response (ypT0: 40.1% vs. 21.6%, *p* < 0.001), node negativity (ypN0: 66.7% vs. 58.4%, *p* = 0.027), and favorable tumor regression grades (PTTRG 0–1: 63.5% vs. 31.4%, *p* < 0.001; LNTRG 0/4: 79.4% vs. 66.0%, *p* < 0.001) (Table 3).

**Table 2:** Inter-observer consistency and prognostic efficacy of various types of TRG

	Mandard	CAP	Becker	JESD	Ryan	Mean value
Kappa value (PT)	0.731	0.782	0.811	0.771	**0.833**	0.785
Kappa value (LN)	0.758	0.777	0.821	0.803	**0.864**	0.804
Mean value	0.744	0.779	0.816	0.787	**0.848**	0.795
PT-TRG	Mandard	CAP	Becker	JESD	Ryan	Mean value
AUC (2year-PFS)	0.573	0.581	**0.601**	0.566	0.499	0.564
AUC (3year-PFS)	0.576	**0.610**	0.592	0.575	0.535	0.577
AUC (4year-PFS)	0.482	0.493	**0.525**	0.496	0.474	0.494
Mean value	0.543	0.561	**0.572**	0.545	0.502	0.545
LN-TRG	Mandard	CAP	Becker	JESD	Ryan	Mean value
AUC (2year-PFS)	0.608	0.613	**0.622**	0.599	0.594	0. 607
AUC (3year-PFS)	0.587	0.584	**0.612**	0.587	0.579	0.589
AUC (4year-PFS)	0.594	**0.596**	0.595	0.583	0.560	0.586
Mean value	0.596	0.597	**0.609**	0.589	0.577	0.594

Bold indicates the maximum value

**Table 3:** Clinicopathological information between different neoadjuvant therapy groups

Variables	Total (*n* = 467)	NCRT (*n* = 282)	NICT (*n* = 185)	*p*	statistic
*Sex, n (%)*				0.493	0.471
Female	67 (14.3)	43 (15.2)	24 (13)		
Male	400 (85.7)	239 (84.8)	161 (87)		
Age, Mean ± SD	60.6 ± 7.2	60.2 ± 7.1	61.2 ± 7.3	0.162	1.957
*Smokeing, n (%)*				0.21	1.575
No	139 (29.8)	90 (31.9)	49 (26.5)		
Yes	328 (70.2)	192 (68.1)	136 (73.5)		
*Drinking, n (%)*				0.873	0.026
No	156 (33.4)	95 (33.7)	61 (33)		
Yes	311 (66.6)	187 (66.3)	124 (67)		
*ypT, n (%)*				< 0.001	Fisher
0	153 (32.8)	113 (40.1)	40 (21.6)		
1	65 (13.9)	36 (12.8)	29 (15.7)		
2	98 (21.0)	56 (19.9)	42 (22.7)		
3	148 (31.7)	74 (26.2)	74 (40)		
4	3 ( 0.6)	3 (1.1)	0 (0)		
*ypN, n (%)*				0.027	Fisher
0	296 (63.4)	188 (66.7)	108 (58.4)		
1	112 (24.0)	68 (24.1)	44 (23.8)		
2	51 (10.9)	24 (8.5)	27 (14.6)		
3	8 ( 1.7)	2 (0.7)	6 (3.2)		
*cT, n (%)*				< 0.001	Fisher
1	2 ( 0.4)	0 (0)	2 (1.2)		
2	23 ( 5.1)	19 (6.7)	4 (2.3)		
3	379 (83.3)	220 (78)	159 (91.9)		
4a	31 ( 6.8)	23 (8.2)	8 (4.6)		
4b	20 ( 4.4)	20 (7.1)	0 (0)		
*cN, n (%)*				0.002	14.99
0	23 ( 5.1)	6 (2.1)	17 (9.8)		
1	147 (32.3)	100 (35.5)	47 (27.2)		
2	227 (49.9)	139 (49.3)	88 (50.9)		
3	58 (12.7)	37 (13.1)	21 (12.1)		
*PTTRG, n (%)*				< 0.001	71.573
0	141 (30.2)	112 (39.7)	29 (15.7)		
1	96 (20.6)	67 (23.8)	29 (15.7)		
2	147 (31.5)	83 (29.4)	64 (34.6)		
3	83 (17.8)	20 (7.1)	63 (34.1)		
*LNTRG, n (%)*				< 0.001	35.434
0	278 (59.5)	170 (60.3)	108 (58.4)		
1	24 ( 5.1)	20 (7.1)	4 (2.2)		
2	36 ( 7.7)	17 (6)	19 (10.3)		
3	61 (13.1)	21 (7.4)	40 (21.6)		
4	68 (14.6)	54 (19.1)	14 (7.6)		

**Fig. 2:**
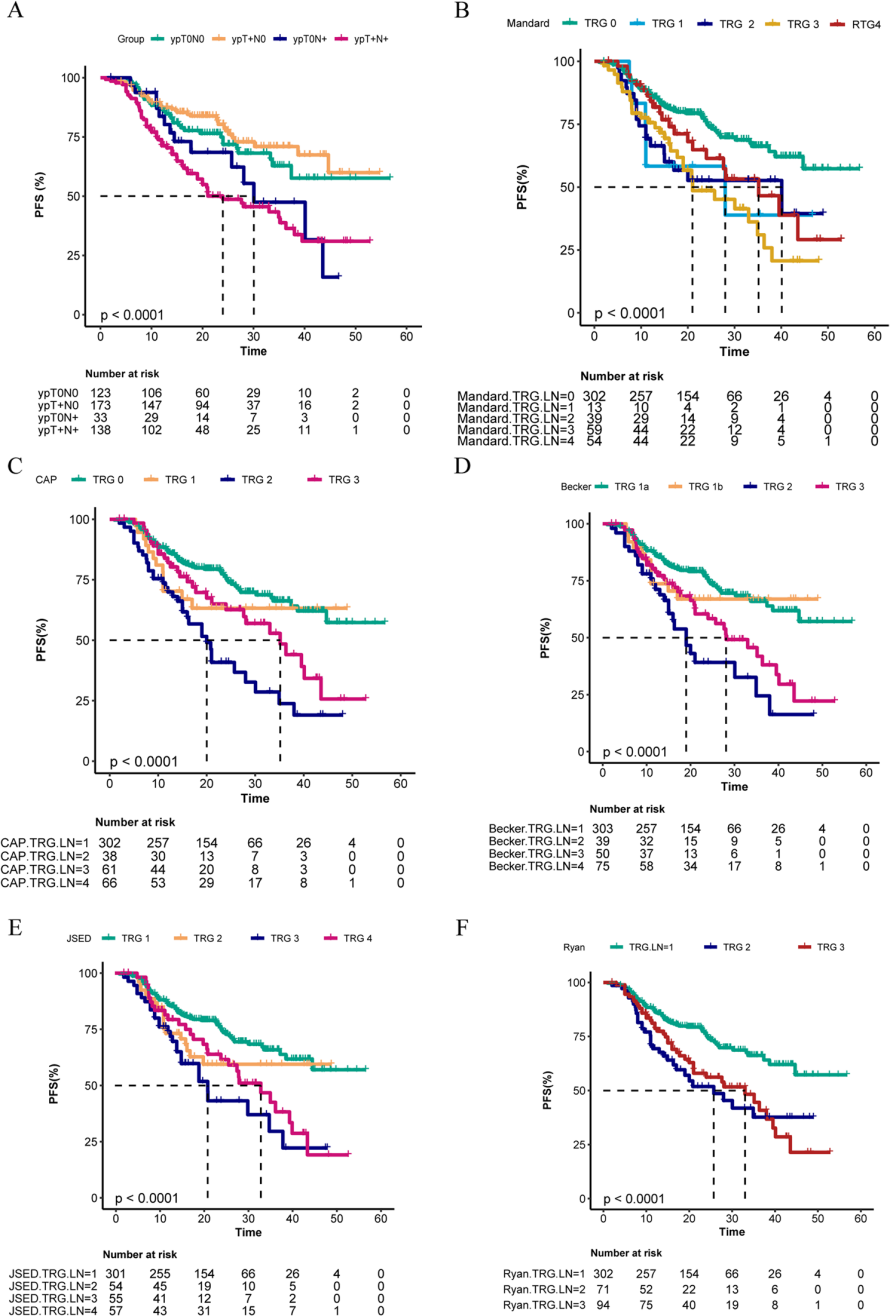
Kaplan-Meier (KM) curves stratifying PFS prognosis based on ypN and ypT (A) and stratification of prognosis using the Mandard, CAP, Becker, JSED, and Ryan standards (B-F)

ROC curve analysis indicated that lymph node regression TRG (LN-TRG) demonstrated higher efficacy in prognostic evaluation compared to primary tumor regression TRG (PT-TRG). Subsequent Kaplan-Meier (KM) curve analysis revealed that ypN (pathological lymph node staging) provided greater value in risk stratification compared to ypT (pathological primary tumor staging) (Figs. 1 and 2A), further confirming the role of lymph node status as a critical factor in prognostic evaluation. Among the five LN-TRG criteria evaluated in this study, all demonstrated some capacity for risk stratification, albeit with limitations. For instance, the Mandard criterion, utilizing a five-category system, could distinctly differentiate TRG0 but performed poorly in distinguishing between TRG1 to TRG4. Similarly, the CAP, Becker, and JSED criteria, which employed a four-category system, exhibited comparable issues, with unclear distinctions between TRG1 and TRG2, as well as TRG3 and TRG4. In certain cases, the distinction between TRG3 and TRG4 even yielded results contrary to expectations. Additionally, the Ryan criterion, which utilized a three-category system, also displayed crossovers within the latter two categories (i.e., higher risk groups) (Figs. 2B-F).

**Fig. 3:**
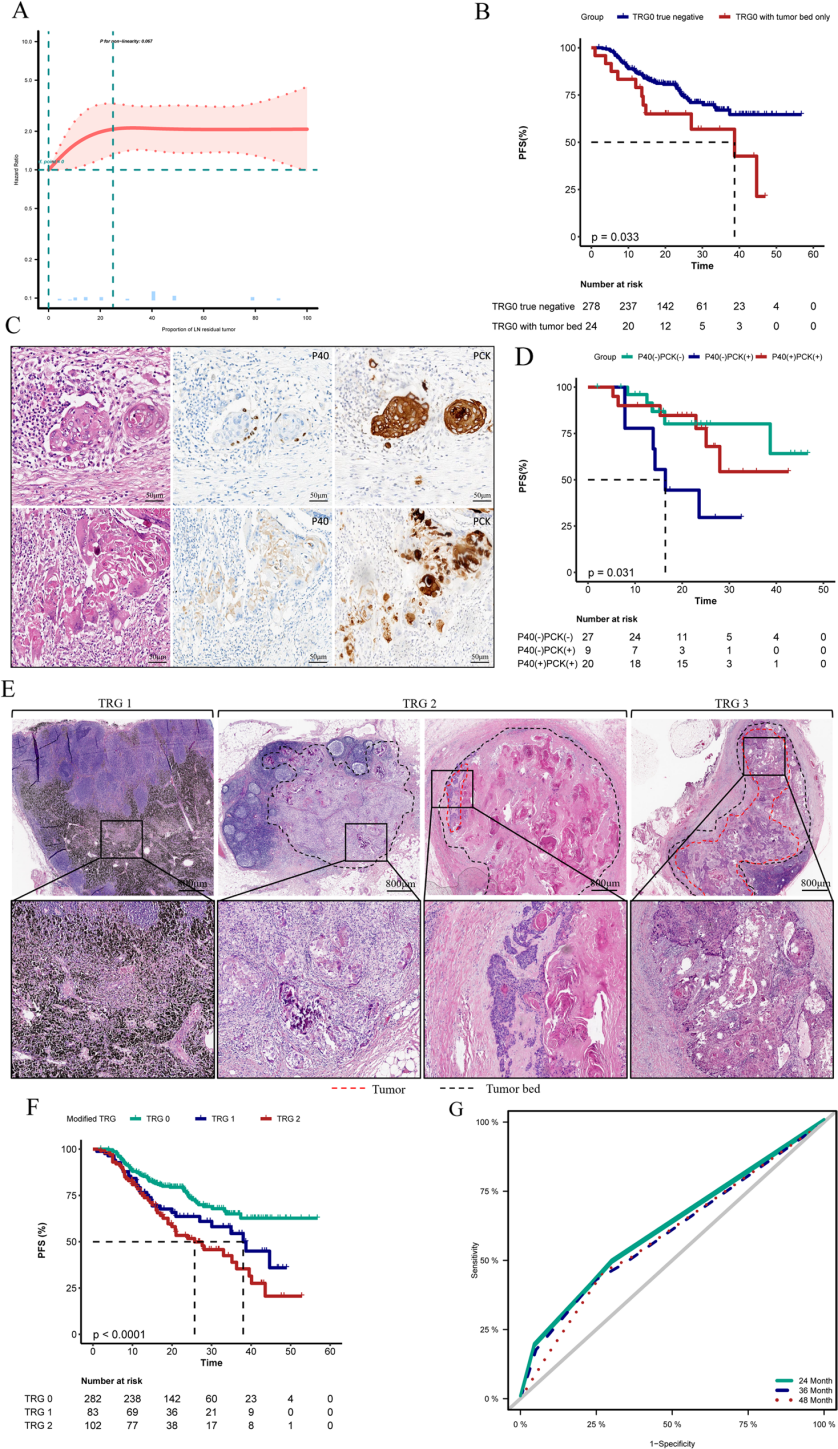
Fitted curves illustrating the relationship between tumor residual proportion and hazard ratio (HR) (A). KM curves demonstrate prognostic differences between residual tumor bed and no residual tumor bed in TRG0 (B). Pathologists utilize immunohistochemistry with P40 and PCK to effectively distinguish the nature of controversial cells (C), and further risk stratification can be achieved through immunohistochemistry (D). Application of the modified TRG assessment standards (E). KM curves show the grouping results of the modified TRG (F). AUC curves illustrate the prognostic predictive efficacy of the modified TRG (G)

Curve fitting analysis allowed us to gain deeper insights into the relationship between tumor residue and prognosis. The results indicated that as the tumor residue ranged from 0 to 25%, the hazard ratio significantly increased with the amount of tumor residue. However, once the tumor residue exceeded 25%, the hazard ratio stabilized and remained high (Fig. 3A). To further enhance predictive efficacy, we conducted a more detailed stratified analysis of TRG0. By comparing lymph node true negatives (i.e., complete absence of tumor residue) with patients exhibiting no cancer residue but with tumor beds, we found that the former had a more favorable prognosis (see Fig. 3B). To improve inter-observer consistency in TRG assessment, we reviewed and analyzed the slides with inconsistent interpretation results. We identified a particular type of cell characterized by eosinophilic cytoplasm and oval nuclei, potentially associated with apoptosis and keratinization. The debate among pathologists centered around whether these cells represented viable tumor cells and whether they should be included in the TRG calculation. To address this issue, we introduced two immunohistochemical markers, P40 and PCK, for differentiation. Specifically, cells that were P40(+) and PCK (+) were classified as viable tumor cells and included in the TRG calculations, while cells that were P40(-) and PCK (+ or -) were regarded as non-viable tumor cells or non-tumor cells (see Fig. 3C). This strategy not only significantly improved inter-observer consistency (Kappa value = 0.921) but also facilitated more accurate prognostic stratification (Fig. 3D).

Based on these findings, we optimized the LN-TRG criteria and employed immunohistochemical techniques to achieve more precise classification. The optimized evaluation criteria are as follows: LN-TRG1 indicates the absence of any tumor or tumor bed residue within the lymph nodes; LN-TRG2 indicates that the tumor residue is between 0% and 25% or there is no tumor but some tumor bed residue; LN-TRG3 indicates that the tumor residue exceeds 25% (see Fig. 3E). The refined LN-TRG criteria allow for more effective risk stratification, exhibiting higher AUC values (0.624) and enhanced consistency coefficients (Kappa value = 0.904) (Figs. 3F and G).

## Discussion

This study systematically assessed tumor regression grade (TRG) in 467 cases of esophageal squamous cell carcinoma (ESCC) patients who received neoadjuvant therapy followed by surgery within a Chinese cohort. We compared the effectiveness and inter-observer consistency of five commonly used TRG evaluation systems: Mandard, CAP, Becker, JSED, and Ryan [10–12], in predicting the prognosis of primary tumors (PT) and lymph nodes (LN) in the Chinese esophageal cancer population. Our findings indicate that the Ryan criteria exhibited the highest inter-observer consistency coefficient, although it had the lowest predictive efficacy. Conversely, the Becker criteria demonstrated a higher predictive performance alongside a good consistency coefficient [11, 12]. The modified LN-TRG system exhibited improved predictive capability and inter-observer consistency.

In the ROC curve analysis, the average AUC value of LN-TRG was significantly higher than that of PT-TRG, directly indicating the superiority of LN-TRG in predicting patient prognosis. Kaplan-Meier (KM) curve analysis further demonstrated that ypN stratified risk more effectively than ypT [13], confirming the important role of lymph node status in prognostic evaluation. Additionally, both PT-TRG and LN-TRG outperformed overall survival (OS) in predicting progression-free survival (PFS), underscoring the potential value of TRG in assessing tumor recurrence. However, it is noteworthy that the predictive efficacy of PT-TRG declined when forecasting long-term PFS (e.g., 4-year PFS), suggesting potential limitations of TRG in long-term recurrence predictions [14].

Among the comparisons of the five assessment standards, we observed that the three-category standard (Ryan) had the highest consistency coefficient, significantly surpassing the five-category standard (Mandard). In terms of predictive efficacy, the standard with a defined cutoff value (Becker) demonstrated higher predictive power and a favorable consistency coefficient. This suggests that assessment systems with fewer categories and clear cutoff values may have distinct advantages. Nevertheless, all five LN-TRG standards have certain limitations in practical application. These TRG standards were initially designed for primary tumor lesions; however, lymph nodes possess unique characteristics and differences [11, 12, 14]. A TRG0 grade may indicate the absence of cancer metastasis but could also imply that cancer metastasis exists but has regressed post-treatment. The KM survival curve analysis revealed distinct prognostic differences between these scenarios, emphasizing the importance of more detailed stratification for TRG0. Moreover, the differentiation between high-grade TRG was suboptimal, as the prognostic distinction between TRG3 and TRG4 was not adequately clear and sometimes counterintuitive.

To identify the reasons for the suboptimal performance, we analyzed the relationship between tumor residuals and prognosis using curve fitting, discovering that when the proportion of residual tumor exceeded a certain threshold, the recurrence risk appeared to stabilize at a high level. Therefore, the value of further distinguishing between high-grade TRG becomes limited. This finding suggests a need to reconsider the threshold effect of tumor residuals in prognostic evaluations rather than averaging them into several categories. To enhance inter-observer consistency in TRG assessments, we introduced immunohistochemical markers P40 and PCK for differentiation. P40 is a nuclear localization marker for squamous cell carcinoma, whereas degenerated cancer cells lose their protein-binding function and subsequently their invasive capability. Utilizing immunohistochemistry allows for better distinction of tumor cell viability and invasive potential [15]. This strategy not only significantly improved inter-observer consistency but also facilitated more precise prognostic stratification.

Based on these findings, we optimized and adjusted the LN-TRG standards by isolating a subgroup with tumor beds from the TRG0 level and reestablishing a 25% cutoff value. Furthermore, we employed immunohistochemical techniques to achieve more accurate classification. The optimized LN-TRG standards exhibited improved performance in risk stratification, with enhancements in both AUC value and consistency coefficient. However, this study does have limitations; the single-center design and lack of external validation may restrict the broader applicability of the modified TRG.

In summary, this study compares the efficacy and inter-observer consistency of different TRG evaluation systems in the prognostic assessment of esophageal cancer. We identified certain shortcomings in each TRG system and proposed adjustments. The modified LN-TRG system demonstrated superior predictive capability and risk stratification effectiveness. These findings may assist clinicians in prognostic assessment in the Chinese esophageal cancer population and provides a critical reference for subsequent precision treatment strategies.

## Electronic supplementary material

Below is the link to the electronic supplementary material.

Supplementary Material 1

## Data Availability

The data are available from the corresponding author on reasonable request. No datasets were generated or analysed during the current study.
