# Supplementary figures and images for: Comparative analysis and optimization of tumor regression grade assessment systems in neoadjuvant therapy for esophageal squamous cell carcinoma
Source: BMC Cancer. 2025 Aug 20;25:1341. doi: 10.1186/s12885-025-14726-4 (PMC12369091; doi:10.1186/s12885-025-14726-4)

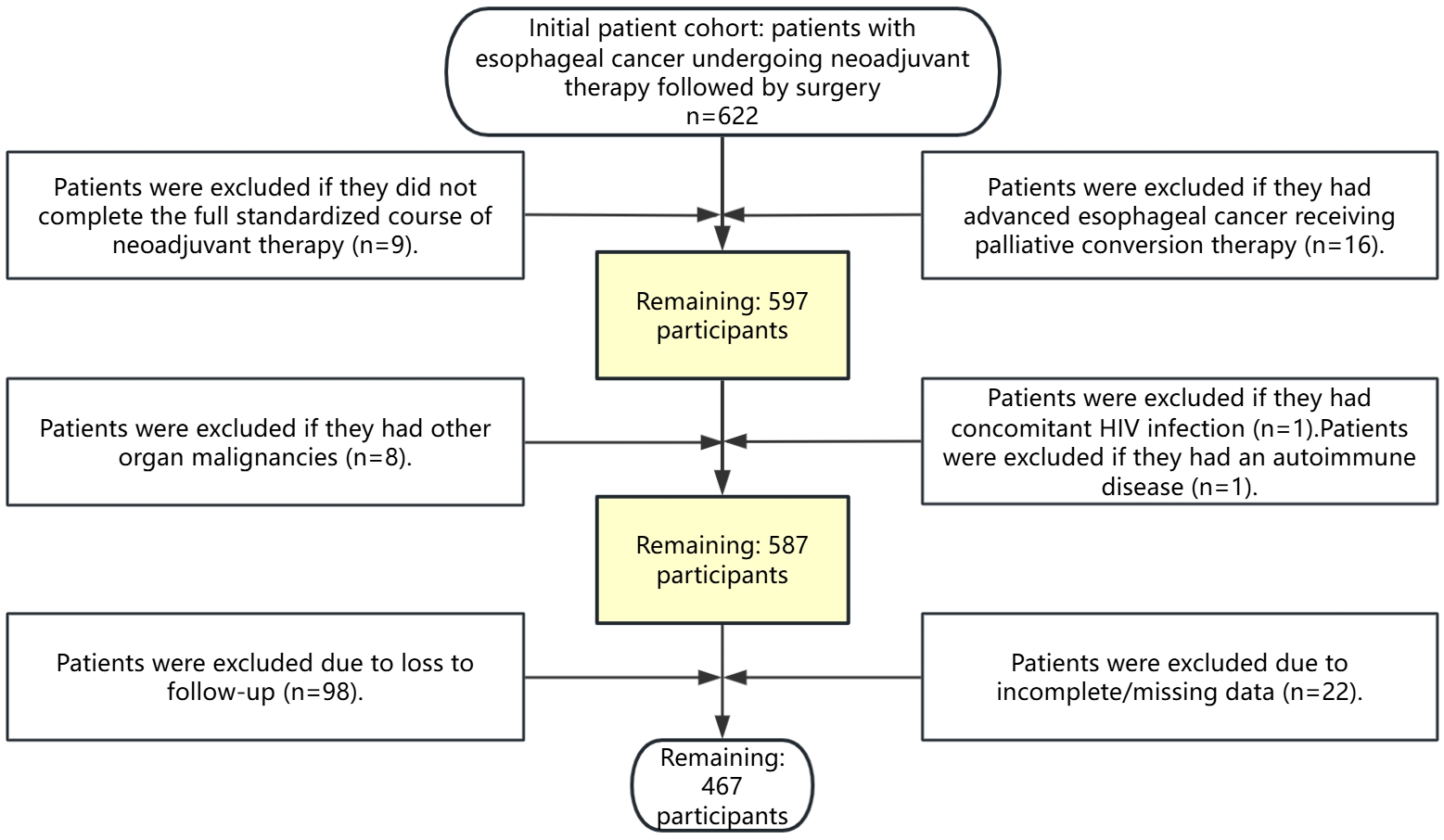

Supplement: Supplementary file 1 — Supplementary Material 1 [file 12885_2025_14726_MOESM1_ESM.png]
